# Operative Treatment of Astigmatism1Read at the Congress of the Société Francaise d’Ophthalmologie, Paris, May, 1896.

**Published:** 1896-08

**Authors:** 

**Affiliations:** Berne


### Translation.

### OPERATIVE TREATMENT OF ASTIGMATISM.[1]

By PROFESSOR PFLÜGER, of Berne.

Translated from *La Clinique Ophthalmologique*, Paris, June, 1896,

By ALVIN A. HUBBELL, M. D., Buffalo, N. Y.

CLINICAL ophthalmometry, developed by Javal, has, by an association of natural ideas, given a glimpse of the possibility of the operative treatment of astigmatism. Traumatic or operative astigmatism consecutive to extraction of the crystalline lens has given form to this idea.

I have considered this question for seven years, and five years ago I presented cases operated upon. I reserve the publication of a further note on this subject, not yet being satisfied with the technique and dosage of the operation. Today, however, I will say a few words, seeing that the same ideas begin to appear elsewhere.

I have been surprised at the changes of curvature of the cornea, following aphakia in eyes strongly myopic (I presented such cases in the form of a table on the occasion of my first communication to this congress: Cessation of strong myopia by aphakia). Every one of us knows sufficiently well that inverse astigmatism ordinarily occurs after operation for senile cataract, the diminution of curvature of the cornea being perpendicular to the incision, and its increase in the direction of this incision, and finally the progressive diminution of this post-operative astigmatism going on from month to month. We know, likewise, that considerable, and often quite regular, astigmatism follows perforating wounds of the cornea, and also ulcerations between the center and limbus without perforation.

Therefore, from these facts, I have begun my trials of correction of astigmatism of the cornea by establishing a loss of substance at the periphery of the cornea, parallel to the limbus, nearly in the region of the arcus senilus. The diminution of the refraction perpendicular to the direction of the wound is made without alteration of the curvature of the cornea in the inverse direction. But the cure is delayed too long and the patients weary of the treatment. For this reason, I have tried to establish a loss of substance of the cornea by the aid of a special "plane" which I

1. Read at the Congress of the Société Francaise d'Ophthalmologie, Paris, May, 1896.

have had constructed with this effect. The idea appeared to be better than its realisation. After several operations, I decided to return to the simple incision of the cornea with the iridectomy knife, having in mind, always, the diminution of the vertical curvature, and less, its augmentation in the direction parallel to the wound.

I make use of some of those rare cases of myopic astigmatism, either of young men desiring to enter military service who are excluded by reason of their astigmatism, or of young women suffering from asthenopia from rather weak degrees of astigmatism.

The correction of hypermetropic astigmatism seems to me to remain a desideratum.

The extraction of the lens in strong myopia, in which I have carefully measured the curvature of the cornea before and after operation, has shown me that, contrary to my ideas, quite often the curvature of the cornea was augmented in all directions.

In the extraction of artificial cataract I have made the incision as much as possible perpendicular to the meridian of greatest refraction. Sometimes, however, I have not followed this rule because of contra-indications which I could not neglect, and this is why the astigmatism is found increased in certain cases in the place of having diminished.

Occasionally, I confess, I have not thought of the correction of the astigmatism. Quite often, moreover, it has not existed. (Here the author presented a table of forty-five cases in which measurements had been taken before and after operation.)

My observations show that in more than one-fourth (twelve) of the cases operated upon, the two principal meridians have gained in refraction ; that in one-fifth (nine) the two meridians have been flattened.

It would be too thankless a task to study very closely the conditions in which an incision of the cornea would effect definitely an augmentation or diminution of the corneal refraction in one meridian or another and to establish a technique of incision of the cornea which will permit one to correct at pleasure a myopic, hypermetropic or mixed astigmatism.

Besides making ophthalmometric measurements before the operation and after it at repeated and prolonged intervals, there should also be considered in these investigations the distance of the corneal incision in relation to the limbus, the size of this incision and its obliquity.

Repeated reopening of the wound would have an influence, probably, on the definite cicatrisation and on the change of the curvature of the cornea.

The age of the patient and the elasticity of the tissues should not be neglected.

As I have shown above, there are other procedures than incision of the cornea to change its curvature. A glimpse is thus given to other methods than those here mentioned capable of producing a change of the cornea with a therapeutic purpose.

It is the coöperation of all our confrères which will aid in shortening the road by which to reach the operative correction of astigmatism.

